# Description and Characterization of Different Types of Lymphoma in Cats in Hong Kong

**DOI:** 10.3390/ani14111654

**Published:** 2024-05-31

**Authors:** Angel Almendros, Long-Ki Chan, Rodrigo dos Santos Horta, Omid Nekouei, Fraser Hill, Antonio Giuliano

**Affiliations:** 1 Department of Veterinary Clinical Sciences, Jockey Club College of Veterinary Medicine and Life Sciences, City University of Hong Kong, Hong Kong; aalmendr@cityu.edu.hk (A.A.); longkchan9@cityu.edu.hk (L.-K.C.); 2 CityU Veterinary Medical Centre, City University of Hong Kong, Hong Kong; 3 Department of Veterinary Clinic and Surgery, Federal University of Minas Gerais, Belo Horizonte 31270-901, MG, Brazil; rodrigohvet@gmail.com; 4 Department of Infectious Diseases and Public Health, Jockey Club College of Veterinary Medicine and Life Sciences, City University of Hong Kong, Hong Kong; omid.nekouei@cityu.edu.hk; 5 VDL Veterinary Diagnostic Laboratory, City University of Hong Kong, Hong Kong; fraser.hill@cityuvdl.com.hk

**Keywords:** feline lymphoma, epidemiology, prevalence, histopathology, cytology, anatomical location, Hong Kong

## Abstract

**Simple Summary:**

Lymphoma is one of the most common neoplastic malignancies in cats. The relative prevalence of different types of lymphoma in cats living in Hong Kong has never been reported. This study provides valuable information on the frequency, characteristics, and anatomical forms of feline lymphoma in cats in Hong Kong, contributing to the limited knowledge of feline lymphoma in Asian populations.

**Abstract:**

This study aimed to characterize and describe the different lymphoma types and anatomical forms in cats in Hong Kong. The clinical and histopathological data of cats diagnosed with lymphoma by cytology and/or histopathology were collected from a large diagnostic laboratory in Hong Kong. In total, 444 cats were diagnosed with lymphoma over four years (2019–2022). Like other countries where there is a low prevalence of FeLV infection, the predominant form of lymphoma was gastrointestinal (abdominal). Nasopharyngeal and peripheral nodal lymphoma were the second and third most common forms of lymphoma. The large cell/high-grade lymphoma type was much more common than the low-grade/small cell lymphoma in the study population. Domestic short hair was the most commonly affected breed in our study (*n* = 259/444). Among the cats with identified T/B-cell status, B-cell lymphoma (*n* = 61/81) prevailed as the most common phenotype. This study describes and characterizes the different types of feline lymphoma in cats in Hong Kong, adding valuable information to the body of knowledge.

## 1. Introduction

Feline lymphoma is a common neoplastic condition that affects cats worldwide. Lymphoma can arise from lymphoid and non-lymphoid tissues throughout the body and can affect various anatomical locations [1,2,3,4,5]. The classification of lymphoma in cats is complex, with different subtypes identified based on their biological behavior, cell origin, and anatomical location [2,6,7]. 

Previous studies have reported variations in the prevalence of different anatomical forms of lymphoma in feline populations across different countries [2,3,5]. Classification based on anatomical location usually differentiates gastrointestinal or alimentary lymphoma from other extranodal forms (nasopharyngeal, renal, central nervous system, etc.), and also multicentric (peripheral lymph nodes), mediastinal, and mixed forms [2,6,7]. The prevalence of one anatomical form of feline lymphoma versus others is largely influenced by the FeLV status [2,3,8,9,10]. Cats infected with FeLV are more likely to develop multicentric, mediastinal, and renal lymphoma [9,10,11]. Also, the age at presentation is mostly influenced by the virus, and cats with persistent FeLV viremia usually develop lymphoma at younger ages than FeLV-negative cats [2,3,10,11]. 

With the widespread use of FeLV vaccination, FeLV-induced forms of lymphoma are now less common [4,7,10]. This leads to regional variability of anatomical presentations with gastrointestinal lymphoma being the most prevalent in countries with a low FeLV prevalence [4,12,13,14], and multicentric and mediastinal forms being more common in countries with a higher rate of FeLV infections [3,15]. Different forms and types of lymphoma have, however, been reported in countries with a relatively low prevalence of FeLV-infected cats such as Australia, Japan, the USA, and other European countries [2,5,16,17,18]. In Hong Kong, FeLV infection is considered rare, similar to what has been reported in the UK and USA [19]. Variable relative prevalences of high-grade and low-grade lymphoma are also reported in different countries and multiple factors may contribute to them, such as breed prevalence, genetic pools, or diagnostic sampling techniques [16,20,21]. As such, the prevalence of low-grade and high-grade lymphoma could be affected by evaluating only samples taken by cytology or histopathology [21]. Cats with high-grade lymphoma usually present with intestinal masses often diagnosed by FNA and cytology, and epidemiological studies including only cytological samples may overestimate the prevalence of large-cell gastrointestinal lymphoma. On the contrary, low-grade/small cell lymphoma rarely forms masses and cytology is not an efficient method for such a diagnosis, which requires histopathology and immunohistochemistry [21,22]. For the same reason that cytology could overestimate the prevalence of large cell lymphoma, samples coming only from histopathology specimens are likely to overestimate the prevalence of low-grade lymphoma. 

To date, there is limited research on frequency and types of feline lymphoma in Asia. Studies investigating the relative prevalence of the different anatomical forms and types of feline lymphoma, with diagnoses obtained by both cytology and histopathology, have not yet been performed in Hong Kong. 

Feline lymphoma subtype distribution in Hong Kong is discussed in this study with the hope of providing a better understanding of its epidemiology that may lead to improving prevention strategies and to an overall better management of the disease. A descriptive analysis of different anatomical forms, grades, and immunophenotypes of feline lymphoma in Hong Kong is therefore presented here.

## 2. Materials and Methods

### 2.1. Data Collection

Data for this study were obtained from the Veterinary Diagnostic Laboratory of City University in Hong Kong (VDL). This is the only laboratory offering clinical pathology and histopathology services that is based in Hong Kong and all analyzed samples were from cats from Hong Kong, submitted from different clinics and hospitals around the Hong Kong SAR. 

The study period spanned four years from January 2019 to December 2022. The electronic database of VDL was searched for all available records on feline lymphoma diagnoses during the study period. Clinical and histopathology PDF file reports for each selected cat were collected and reviewed. Only cases with a confirmed diagnosis of lymphoma by cytology and/or histopathology were included in the final data analysis and all the cases included were diagnosed by a certified veterinary pathologist. When the diagnosis was not confirmed, further immunohistochemistry or immunocytochemistry were often used. Immunohistochemistry and immunocytochemistry were performed with standard antibodies; CD-3 for T-cell origins and PAX-5 for B-cell origins. In specific cases, other antibodies were used to rule out other round-cell neoplasia at the discretion of the pathologist.

When a case had multiple follow-up reports or additional tests, such as immunohistochemistry or immunocytochemistry, only the most comprehensive report with the first lymphoma diagnosis was included. Where both histopathology and cytology reports were available for a single diagnosis event, the final histopathology report was included in the analysis. Information collected from the PDF files included the breed, age, location of the lymphoma, grade, and B- or T-cell immunophenotype when available.

The anatomical form of lymphoma was categorized according to the previously published literature [2,18,23]. Specific forms of lymphoma, including gastrointestinal (GI), nasal/nasopharyngeal, renal, laryngeal, CNS, eye, and skin, were considered separately, but were also grouped into abdominal and thoracic, when diagnosed inside the abdomen or the thorax, respectively. Multicentric lymphoma was considered a form of lymphoma involving peripheral lymph node/s, affecting multiple locations. Unusual lymphoma presentations, which did not fall into any of the above categories, were categorized as “other”.

### 2.2. Statistical Analysis

All statistical analyses were conducted in Stata v18 (StataCorp LLC, College Station, TX, USA). Descriptive statistics for signalment and anatomical locations for selected cases were generated. The potential associations of breed (categorized as DSH or pure) and cell size (large or small) with the anatomical location (GI tract or not) of lymphoma were evaluated using Chi-square tests. To investigate the potential association between sex and lymphoma in our study population, the male proportion of our cases was compared with the male proportion of all admitted cats to the Veterinary Medical Centre of City University of Hong Kong (VMC) during the study period using the test of proportions. 

## 3. Results

A total of 444 cats were included in our study based on the inclusion criteria (i.e., confirmation of feline lymphoma by a certified pathologist). There were 258 males (58%) and 186 females (42%). The sex ratio among our lymphoma cases (male proportion = 58%) was not significantly different from the expected sex ratio of cats admitted to one of the largest veterinary hospitals, Veterinary Medical Center (VMC) (male proportion = 56.8%), indicating no association between sex and lymphoma in our study (*p* = 0.408). Of the 444 cats, 365 (82.2%), including 213 males and 152 females, were neutered. There were 26 different breeds in the final dataset, with domestic shorthair (DSH) (58%; 259/444) and British shorthair (BSH) (20%; 88/444) being the most common. There was no statistically significant association between the breed (being pure or not) and GI location of lymphoma in the cases studied (*p* = 0.372). The age of the cats ranged between 6 months and 21 years (media *n* = 10 years). Among the 444 cats with lymphoma, 133 were diagnosed by histopathology (9 of which also had cytology results) and 311 by cytology alone.

Large cell/high-grade lymphoma was the most common subtype (84.7%; 376/444). Small cell/low-grade lymphoma accounted for 9.7% of cases (43/444), and 25 cases (5.6%) lacked recorded cell grade or size information (Table 1). Of the 376 large cell/high-grade lymphoma cases, 286 (76%) were diagnosed with cytology and 90 (24%) with histology. Of the 43 small-size/low-grade lymphoma cases, 17 (39%) were diagnosed with cytology and 26 (61%) with histopathology. 

Among the 81 cats with T/B-cell status identified with both immunohistochemistry or immunocytochemistry, B-cell lymphoma was the most prevalent (75.3%), followed by T-cell lymphoma (17.3%) and non-B-non-T lymphoma (3.7%) (Table 2). Of these 81 cats, 34 cases (42%) were abdominal lymphoma (26 B-cell, 7 T-cell, and 1 non-B-non-T), 25 nasopharyngeal lymphoma (23 B-cell and 2 T-cell), and 6 multicentric nodal lymphoma of the neck (5 B-cell, 1 T-cell). Mixed lymphoma and “other” categories included four cases. Three cases were multicentric lymphoma (two non-B-non-T, one T-cell) and two were skin lymphoma (one for T-cells and B-cells each). Each case of laryngeal lymphoma and leukemia was T-cell-based and one lymphoma case of the eye was B-cell-based. 

**Figure 1:**
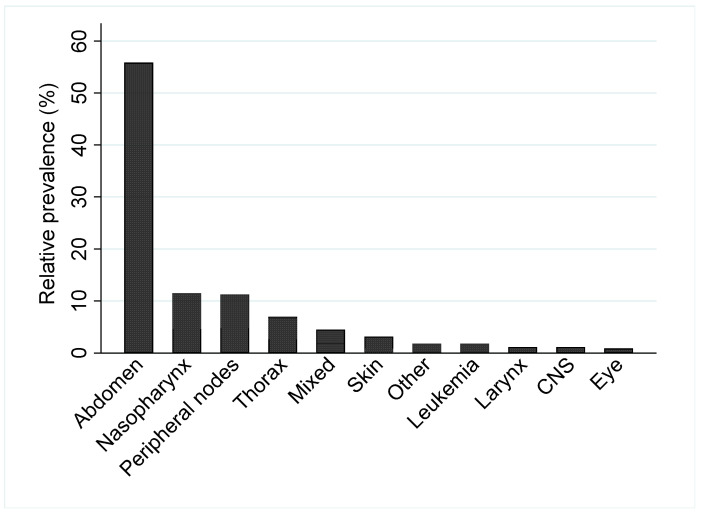
Relative prevalence of 444 feline lymphoma cases by main anatomical locations.

Among the diagnoses made by both cytology and histopathology, abdominal lymphoma was the most prevalent with 248 cases (55.8%), followed by 51 cases of nasopharyngeal lymphoma (11.5%), 50 infiltrating peripheral lymph nodes (11.3%), 31 thoracic lymphomas (7%), 20 mixed lymphomas (4.5%), 14 cutaneous lymphomas (3.2%), 8 leukemias (1.8%), and 5 CNS lymphomas, all sampled from cerebrospinal fluid cytology (1.1%); 5 laryngeal lymphomas (1.1%); and 4 ocular lymphomas (0.9%). Eight cases (1.8%) did not fall into any of the above categories and were thus allocated into “other” (Figure 1). Table 3 summarizes the distribution of 444 lymphoma cases based on the histopathology versus cytology diagnosis in different anatomical forms.

Abdominal lymphomas included cases affecting the gastrointestinal tract (*n* = 155), kidney (*n* = 28), mesenteric lymph nodes (*n* = 9), liver (*n* = 8), and spleen (*n* = 3). About 9.3% (*n* = 23) of abdominal lymphomas were found in multiple abdominal locations, rather than a single location, and 13 cases (5.2%) did not have detailed locations except for an abdominal origin, while 9 cases (3.6%) of abdominal lymphoma were diagnosed from peritoneal effusion. Of the 155 cases of a gastrointestinal origin, only 1 lacked specific GI location information. The stomach was affected in 55 cases, and 97 cases were intestinal, in which the small intestine accounted for 43 cases with 18 cases specifically recorded in the jejunum, 7 in the ileum, 2 in the duodenum, 3 in both the duodenum and jejunum, 1 in both the ileum and mesenteric lymph nodes, and 1 in the jejunum/ileum and unspecified lymph nodes. Twelve cases affected the colon, one the caecum, and two the rectum, contributing to a total of fifteen cases (9.7% of all gastrointestinal ones) in the large intestine. The remaining 39 intestinal cases (25.2% of all gastrointestinal ones) were affecting the ileocaecocolic junction (11 cases) and the lymph nodes (9 cases) or were unspecified (19 cases) (Table 4).

The second most common category in this study, nasopharyngeal lymphoma, affected the nasal cavity in 51 cases (15.6%).

Multicentric lymphoma was the third most common category (50 cases, 11.3% of all lymphomas) and involved mainly lymph nodes of the neck, of which 23 cases (46%) were neck lymphomas, 15 cases (30%) affected only the mandibular or submandibular lymph nodes, 4 cases (8%) affected only the retropharyngeal lymph nodes, and 1 case was not specified. Other lymph nodes affected included one case of inguinal lymph nodes. Six cases accounting for 12% of peripheral nodal lymphomas were multicentric, involving multiple lymph nodes, for example, one case with a combination of axillary, popliteal, and neck lymph nodes; one case with a combination of pre-scapular and axillary lymph nodes; one case of both mandibular lymph and popliteal lymph nodes; one case of popliteal lymph nodes; and one case of the pre-scapular and abdominal lymph nodes.

Thoracic lymphoma accounted for 7% of all lymphomas. Out of 31 cases, 5 cases (16.1%) were from the mediastinum, 4 cases (12.9%) were from pulmonary parenchyma, and 3 cases were not specified (9.7%). More than half of the cases (19 cases, 61.3%) were non-specified and diagnosed from the pleural effusion. 

Mixed forms were present in 20 cases, 12 cases (60%) of which were thoracic and abdominal. The combination of nodes of the neck with thoracic or abdomen sites (3 cases, 15%) and nasal cavity with oral or neck sites (3 cases, 15%); 1 case of subcutaneous and rectal involvement; and 1 case of abdominal and mammary chain involvement represented the remaining combinations in the mixed form lymphoma.

Fourteen cases were cutaneous lymphoma, of which thirteen cases had location information available. Notably, the limb was the most affected area (five cases, 35.7%), including three cases attributed to the hindleg or hock, one case to the carpus, and one case to the paw. Specific occurrences were recorded in the scapular region, the skin of the thorax, and the anus, representing one case each. Additionally, 21.4% were reported as head-related locations, and 14.3% involved multiple locations, of which one sample was from the head and paw, and another sample was from the ear and neck.

Eight cases did not fit into any predefined category and were called “others”. Among these “others,” a significant majority (5 cases, 60%) were found to originate from various parts of the oral cavity. Specifically, two cases arose from the tongue, two cases from the hard palate, and one case from the maxilla. A total of 25% of the eight cases were related to the trachea, and one case was sampled from the mammary gland, contributing to the diversity of anatomical sites within this group.

Certain specific types of lymphoma, in particular large granular lymphoma (LGL), were diagnosed in 17 cases (3.8% of all lymphomas). Ten cases of LGL were abdominal (58.8%), most affecting the GI tract. Three cases (17.6%) were thoracic. Laryngeal lymphoma, inguinal lymph nodes, mixed lymphoma, and subcutaneous lymphoma each accounted for one LGL case. Cases of Hodgkin lymphomas were not reported in the final diagnosis by cytology nor histopathology; Hodgkin lymphoma was suggested as possible in four cytology cases and histopathology was not available for those cases for confirmation. All four cases were from the lymph nodes of the neck, and one of those also presented gastrointestinal involvement. Non-B and non-T lymphomas were reported in three cases, two from cytology and one from histopathology.

A total of eight cases were diagnosed as leukemia, of which chronic or small cell leukemia and acute large cell leukemia accounted for half each. The specific types of leukemia included Chronic Lymphocytic Leukemia (CLL) (4 cases, 50%) and Acute Lymphoblastic Leukemia (ALL) (3 cases, 37.5%). All were diagnosed from peripheral blood smears and two cases each were also diagnosed from bone marrow. One acute leukemia case was suspected based on cell morphology revealed to be Acute Myeloid Leukemia (AML) identified and sampled from the peripheral blood and spleen.

## 4. Discussion

The findings of this study corroborate previous similar research conducted in other countries but also highlight some potential differences in lymphoma characteristics affecting cats in Hong Kong and possibly in other regions of Asia compared to Western countries. Sex predisposition to feline lymphoma is controversial with some studies showing no sex predisposition [4,24], while others suggest a possible increase in incidence in males compared to females [1,2,10,13,14]. In our study, more males were diagnosed with lymphoma compared to females. However, once we compared the male–female distributions in the patients presented to one of the largest hospitals in Hong Kong, the difference was not statistically significant. Further investigations are warranted to investigate if there is a sex predisposition for lymphoma in cats.

The age distribution of lymphoma cases in this study mirrors findings from other studies, with a peak incidence observed around 10 years of age, confirming that in Hong Kong, middle-aged/older cats are the most predisposed [1,2,18]. 

The prevalence of large cell/high-grade versus small cell/low-grade lymphoma in cats is rarely reported [1,16,21]. The predominance of large cell/high-grade lymphoma in our study is consistent with a previous small study conducted from three veterinary practices in Australia, suggesting that feline lymphoma often presents as an aggressive malignancy [21]. In another single-referral-center study, when the author analyzed all the gastrointestinal lymphomas referred to the hospital, large cell lymphoma was slightly more common than small cell lymphoma [25]. Again, similar to our findings, in the largest study conducted in insured cats in the UK, large cell/high-grade lymphoma was much more common than small cell/low-grade lymphoma [1].

Despite some studies suggesting that the small cell/low-grade form of lymphoma is the most common type of feline lymphoma, these studies are biased by the analysis of only histopathology samples. The majority of large cell lymphomas are diagnosed mainly by cytology [3,16,21]. As an example, in the study by Leite-Filho et al. conducted in Brazil, despite the high prevalence of FeLV-positive cats, low-grade lymphoma was the most common, which is different from other studies conducted in Brazil where there is still a high prevalence of FeLV-positive cats and most diagnoses are made by cytology [15,26]. This is most likely because in the study of Leite-Filho, only cases diagnosed by histopathology were included, biasing the result in favor of low-grade lymphomas that are mostly diagnosed by histopathology versus high-grade lymphomas that are diagnosed prevalently by cytology. 

The low number of confirmed B- or T-cell phenotypes (81 cases, 18%) is probably due to a high percentage of cases being diagnosed by cytology. Another reason could be that T- and B-cell phenotypes are not considered of prognostic relevance in cats and phenotyping is rarely needed to confirm the diagnosis of large cell lymphoma [11,25,27]. The identification of B-cell lymphoma as the most common subtype aligns with previous studies [12,23], and this correlates with the fact that the most common form of lymphoma, the alimentary large cell lymphoma, is usually of a B-cell origin [23].

The higher frequency of abdominal lymphoma, especially GI lymphoma observed in this study, is consistent with studies from the USA, the United Kingdom, and other European countries where there is a similar low prevalence of FeLV, and for the same reason, multicentric, mediastinal, and other forms of lymphoma are rarer [1,9,10,11,12,13,19]. Interestingly, a form of nodal lymphoma affecting the cervical region was the third type of the most common lymphoma in Hong Kong. This is similar to what has been published in a smaller study in Australia, in which neck lymphoma was the third most common form of lymphoma [2,28]. This has been rarely reported in other countries where a localized form of neck lymphoma is likely to be a Hodgkin-type lymphoma [29].

All the peripheral nodal neck lymphomas that were diagnosed by histopathology in our lab were not Hodgkin-like lymphomas; interestingly, four were suspected by cytology but never confirmed with histopathology. There were also three reports with non-B-non-T status but none of those three cases overlapped with the four possible Hodgkin-type lymphoma cases. Most of the cases of nodal neck lymphoma diagnosed by histopathology were predominately diffuse large B-cell lymphomas, while the ones diagnosed by cytology were also large B-cell lymphomas.

The relatively higher frequency of “neck lymphoma” reported here and in the previously reported study in Australia has never been confirmed in other studies [1,2,29]. This could represent a real difference in lymphoma presentation in Australia and Asia compared to other countries or just be a bias from the retrospective study design. An explanation could be that other enlarged peripheral lymph nodes were not felt, reported, or sampled by the veterinarian, and the “neck lymphoma” could just represent a form of multicentric lymphoma; however, this form is still rare in regions with low incidences of FeLV, including Hong Kong (0.2%) [1,10,19].

The real incidence of leukemia in cats is unknown, but while acute lymphoid leukemia seems to be the most common type in cats with persistent FeLV viremia, most reports of acute or chronic leukemia in cats are limited to a single or few cases reports [30,31]. As the incidence of FeLV is low in Hong Kong, it is suggested that most of the cases were chronic lymphoid leukemia. Although there is no consensus in veterinary medicine, the World Health Organization suggests a cut-off point of 20% of blasts in the peripheral blood or bone marrow for the diagnosis of acute leukemia and 15% of any mature cell lineage, in the bone marrow, for the diagnosis of chronic leukemia [32]. A definitive diagnosis should be made based not only on morphology but immunophenotyping as well [29]. However, flowcytometry for immunophenotyping is rarely performed in cats due to the cost and availability of laboratories with validated antibodies, and because it is unlikely to change the prognosis and treatment once chronic (small cells) and acute (large cells) circulating cells are diagnosed on the blood smear. From our data, all the diagnoses of leukemia were made by morphological evaluation, and this is also likely due to the lack of commercially available flow cytometry in Hong Kong.

Large granular lymphocyte lymphoma (LGL) is an uncommon variant of feline lymphoma, morphologically distinct, and easily diagnosed with cytology [33]. It is a very aggressive form of lymphoma, which is more often diagnosed in the gastrointestinal tract and mesenteric lymph nodes, but can simultaneously affect other organs, and in up to 91.7% of cases, may infiltrate the liver, spleen, and kidney, without compromising the gastrointestinal tract [33]. Bone marrow can also be involved with or without hematological abnormalities [33]. It is an extremely aggressive type of lymphoma with a median overall survival time of only 20 days [34]. Only 3.8% of lymphomas were the LGL type, and this is similar to what has been reported before, as LGL is a rare type of lymphoma/leukemia in cats; however, the relative prevalence of this particular type of lymphoma has rarely been reported [33,35].

There are a few limitations of this study. Due to the retrospective nature of this study, the analysis of the cytology and histopathology reports could have biased the results. Similarly, due to data privacy access limitations, the lack of full clinical history and records could also have biased some of the results. For example, a clinical record of lymph nodes affected and classified as only neck involvement could be indeed a misclassification of a multicentric or a mixed form, due to an inaccurate staging. For the same reason, some advanced lymphomas with bone marrow infiltration could have been misdiagnosed as leukemia. Most of the patients with small cell lymphoma did not have PARR testing and this could have biased some of the results too. 

Another limitation is the collection of data from only one veterinary laboratory (VDL) that might not be fully representative of the Hong Kong population of cats, therefore being unable to report true prevalence figures. Other veterinarians in Hong Kong might send samples for histopathological interpretation abroad, hence reducing the accuracy of reporting the regional prevalence. A lack of available data on age and breed distribution was another limitation in this study. A way to overcome these limitations might be by collaborating and sharing data with other clinics in Hong Kong. Further research including prospective study designs is recommended. 

## 5. Conclusions

This study provides a valuable descriptive analysis on the relative frequency of different anatomical forms and types of diagnosed feline lymphomas in Hong Kong, contributing to the limited body of knowledge on feline lymphoma in the region. Further research and collaborative studies across different laboratories and countries in Asia could further enhance our understanding of the epidemiology and etiology of feline lymphoma on a large global scale.

## Figures and Tables

**Table 1:** Frequency distribution of large and small cell lymphoma cases by different anatomical forms of lymphoma.

	Cell Size/Grade			
Main Location	Large/High Grade (Number, % of Cases from the Same Location)	Small/Low Grade (Number, % of Cases from Same Location)	Not Provided (Number, % of Cases from the Same Location)	Total (Number, % of All Cases)
Gastrointestinal (GI)	125 (80.7%)	27 (17.4%)	3 (1.9%)	155 (34.9%)
Abdomen (non-GI)	60 (92.3%)	5 (7.7%)	0 (0%)	65 (14.6%)
Multicentric	46 (92%)	3 (6%)	1 (2%)	50 (11.3%)
Nasopharynx	41 (80.4%)	2 (3.9%)	8 (15.7%)	51 (11.5%)
Kidney	28 (100%)	0 (0%)	0 (0%)	28 (6.3%)
Thorax	26 (83.9%)	1 (3.2%)	4 (12.9%)	31(7%)
Mixed	19 (95%)	0 (0%)	1 (5%)	20 (4.5%)
Skin	10 (71.5%)	1(7.1%)	3 (21.4%)	14 (3.2%)
Leukemia	4 (50%)	4 (50%)	0 (0%)	8 (1.8%)
Other	6 (75%)	0 (0%)	2 (25%)	8 (1.8%)
Larynx	5 (100%)	0 (0%)	0 (0%)	5 (1.1%)
CNS	3 (60%)	0 (0%)	2 (40%)	5 (1.1%)
Eye	3 (75%)	0 (0%)	1 (25%)	4 (0.9%)
Total	376 (84.7% of all cases)	43 (9.7% of all cases)	25 (5.6% of all cases)	444 (100%)

**Table 2:** T- and B-cell lymphoma diagnosed with cytology versus histopathology.

T-Cell/B-Cell Phenotype	Cytology	Histopathology	Total
B-cell	10	51	61
T-cell	7	7	14
T-cell-rich, B-cell	0	1	1
non-B, non-T	2	1	3
T-cell-negative	1	0	1
B-cell with reactive T-cell infiltration	0	1	1
Not provided	291	72	363
Total	311	133	444

**Table 3:** Frequency distribution based on report type and anatomical form of lymphoma.

Location	Cytology	Histopathology	Total
Gastrointestinal (GI)	100	55	155 (34.9%)
Abdomen (other)	63	2	65 (14.6%)
Kidney	26	2	28 (6.3%)
CNS	5	0	5 (1.1%)
Eye	2	2	4 (0.9%)
Larynx	4	1	5 (1.1%)
Leukemia	8	0	8 (1.8%)
Mixed	15	5	20 (4.5%)
Nasopharynx	8	43	51 (11.5%)
Other	3	5	8 (1.8%)
Peripheral lymph nodes	40	10	50 (11.3%)
Skin	8	6	14 (3.2%)
Thorax	29	2	31 (7%)
Total	311	133	444 (100%)

**Table 4:** Different anatomical locations of abdominal lymphomas.

Location	Total
Gastrointestinal (GI)	155
Kidney	28
Mesenteric lymph nodes	9
Liver	8
Spleen	3
Multiple locations	23
Unspecified	13
Effusion	9
Total	248

## Data Availability

The data presented in this study are available on request from the corresponding author. The data are not publicly available due to the confidentiality preferences of the collaborating laboratory.
